# A Novel Three-Dimensional Approach Towards Evaluating Endomyocardial Biopsies for Follow-Up After Heart Transplantation: X-Ray Phase Contrast Imaging and Its Agreement With Classical Histopathology

**DOI:** 10.3389/ti.2023.11046

**Published:** 2023-01-24

**Authors:** Ivo Planinc, Ivana Ilic, Hector Dejea, Patricia Garcia-Canadilla, Hrvoje Gasparovic, Hrvoje Jurin, Davor Milicic, Bosko Skoric, Marco Stampanoni, Bart Bijnens, Anne Bonnin, Maja Cikes

**Affiliations:** ^1^ Department of Cardiovascular Diseases, University Hospital Centre Zagreb, University of Zagreb School of Medicine, Zagreb, Croatia; ^2^ Department of Pathology and Cytology, University Hospital Centre Zagreb, University of Zagreb School of Medicine, Zagreb, Croatia; ^3^ Paul Scherrer Institute (PSI), Villigen, Switzerland; ^4^ Institute for Biomedical Engineering, University and ETH Zurich, Zurich, Switzerland; ^5^ Institut de Recerca Biomèdica August Pi i Sunyer (IDIBAPS), Barcelona, Spain; ^6^ BCNatal-Barcelona Center for Maternal-Fetal and Neonatal Medicine, Hospital Sant Joan de Déu and Hospital Clínic, University of Barcelona, Barcelona, Spain; ^7^ Cardiovascular Diseases and Child Development, Institut de Recerca Sant Joan de Déu, Esplugues de Llobregat, Spain; ^8^ Department of Cardiac Surgery, University Hospital Centre Zagreb, University of Zagreb School of Medicine, Zagreb, Croatia; ^9^ Catalan Institution for Research and Advanced Studies (ICREA), Barcelona, Spain

**Keywords:** heart transplantation, synchrotron, histopathology, graft rejection, X-ray phase contrast imaging

## Abstract

Endomyocardial biopsies are the gold standard for surveillance of graft rejection following heart transplantation, and are assessed by classical histopathology using a limited number of previously stained slices from several biopsies. Synchrotron propagation-based X-ray phase contrast imaging is a non-destructive method to image biological samples without tissue preparation, enabling virtual 2D and 3D histopathology. We aimed to show the feasibility of this method to assess acute cellular rejection and its agreement to classical histopathology. Right ventricular biopsies were sampled from 23 heart transplantation recipients (20 males, mean age 54±14 years) as part of standard follow-up. The clinical diagnosis of potential rejection was made using classical histopathology. One additional study sample was harvested and imaged by X-ray phase contrast imaging, producing 3D datasets with 0.65 μm pixel size, and up to 4,320 images per sample. An experienced pathologist graded both histopathological and X-ray phase contrast images in a blinded fashion. The agreement between methods was assessed by weighted kappa, showing substantial agreement (kappa up to 0.80, *p* < 0.01) between X-ray phase contrast imaging and classical histopathology. X-ray phase contrast imaging does not require tissue processing, allows thorough analysis of a full myocardial sample and allows identification of acute cellular rejection.

## Graphical Abstract

**Figure:**
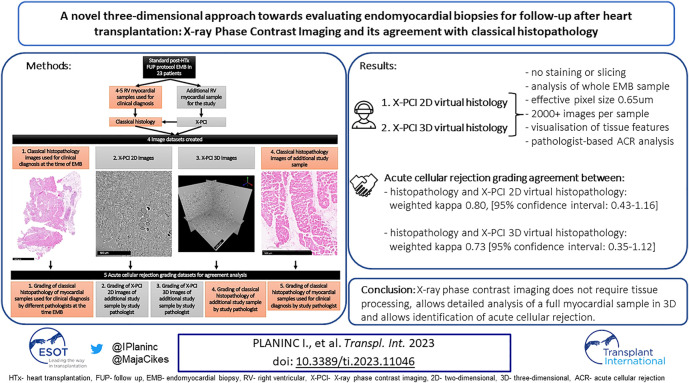
A novel three-dimensional approach towards evaluating endomyocardial biopsies for follow-up after heart transplantation: X-ray Phase Contrast Imaging and its agreement with classical histopathology.

## Introduction

Close monitoring and follow-up of heart transplantation (HTx) recipients is essential for timely recognition of post-transplantation complications, such as acute cellular rejection (ACR) (1, 2). Echocardiography or cardiac magnetic resonance imaging are powerful in the detection of global and regional cardiac dysfunction allowing for indirect identification of fibrosis, but are unable to specifically diagnose rejection (3). “Liquid biopsies” based on cell-free DNA technology are emerging tools in recognition of ACR, however still without wide acceptance in everyday clinical practice (4). Therefore, histopathological analysis of endomyocardial biopsy (EMB) samples remains the standard of care in rejection surveillance (5).

Synchrotron radiation-based X-ray Phase Contrast Imaging (X-PCI) has become a well-accepted technique in soft tissue research. In X-PCI, advantage is taken of the refractive properties of X-rays when traveling through soft tissues to increase the contrast of resulting images. Given the need for highly coherent X-ray beams, synchrotrons (large scale research facilities) are currently primarily used for X-PCI, providing three-dimensional (3D) high resolution (<1 µm pixel size) imaging with excellent contrast.

In cardiovascular research, X-PCI has so far been utilized to study heart architecture *ex-vivo* in animal models, and human hearts (fetal and adult), both healthy and diseased (6–13).

In this pilot study, we aimed to show the potential of X-PCI to assess features of ACR in full 3D volumes of EMB samples, and its agreement with clinical histopathology.

## Methods

### Patients

We included 23 HTx recipients that underwent scheduled EMBs per Institutional protocol. The first 20 patients were included in consecutive manner, and the remaining three patients with known high-grade rejection (2R or 3R) were included from the Institutional archives to enrich the initial sample that was lacking such findings. The proportion of added high-grade rejection samples was based on the occurrence of graft-rejection requiring treatment of around 7%–10% of surveillance EMBs (2, 14). Patient medical data were collected retrospectively. The study was approved by the institutional Ethics review board (Approval of the Ethics Committee of the University Hospital Centre Zagreb, Croatia; Class: 8.1-17/137-2, No: 02/21 AG), and all of the patients signed an informed consent.

### Endomyocardial Biopsy

The EMB was performed following a standardized clinical procedure, and according to the technical recommendations proposed by ISHLT (Supplementary Material) (4). Besides 3–4 myocardial samples used for histopathological diagnosis in the clinical setting, an additional sample was taken for the purposes of this study; all of the samples were initially placed in formalin solution.

### X-Ray Phase Contrast Imaging Acquisition and Visualization

Synchrotron radiation-based X-PCI acquisition was performed at the TOMCAT beamline of the Swiss Light Source (Paul Scherrer Institute, Switzerland). With no further tissue preparation, the samples (at propagation distance of 20 cm between sample and detector) were fully illuminated by a monochromatic X-ray beam with an energy of 20 keV. X-rays were converted to visible light, amplified by 10x objective, and recorded with an effective pixel size of 0.65 µm. (Supplementary Figure S1) (7). For each tomogram, a total of 2,501 projections, 20 darks and 50 flats were acquired with a exposure time of 200 ms, resulting in approximately 10 min acquisition time followed by approximately 3 min for reconstruction of 3D datasets. Reconstructed 3D datasets were obtained from projections using the Gridrec algorithm (7, 15). Depending on the true size of the imaged myocardial tissue sample, one or several tomograms were made, and X-PCI datasets consisted of 2,160–4,320 images of 1.66 mm × 1.66 mm size and 0.65 µm thickness (Supplementary Material). The whole process of sample positioning, X-ray imaging, and dataset production required approximately 20 min per usual sized EMB (one tomogram per biopsy).

The identification of the histopathological features of ACR on the obtained X-PCI images, as well as image optimization with contrast and brightness adjustment was done using the open-source software Fiji (version of the program: ImageJ 1.51s, Wayne Rasband, National Institute of Health, United States) (16, 17).

### Histopathology

Samples were stained by hematoxylin and eosin (H&E) and fixated on glass slides. At least 10 sections were analyzed by light microscopy for diagnosis of ACR according to ISHLT 2004. Recommendations, while immunohistochemistry was performed as part of the clinical patient management, but not on the study samples for which the focus was on ACR (5).

Classical histopathology was initially done on the 3–4 myocardial samples for routine clinical diagnosis of ACR grade, together with routine immunohistochemistry.

### Research Protocol and Datasets for Comparative Graft Rejection Analysis

For comparative ACR analysis, the X-PCI images were presented in two ways: firstly, X-PCI 2D datasets mimicked the ISHLT recommendations for analysis of classical histopathological EMB samples for ACR (5). From the full 3D dataset originally containing up to 4,320 images, 10 images were randomly selected for analysis, maintaining the original superior-inferior sample orientation. If the distance between the slices was too large to adequately assess histopathological features, additional consecutive images from the 3D dataset were obtained.

Secondly, in the X-PCI 3D dataset, the pathologist could use any of the images (up to 4,320) from the sample (Graphical Abstract).

An experienced pathologist assessed ACR grades in both histopathological and X-PCI images, blinded to the identity and any clinical data of the patient. The same pathologist also re-assessed the original samples harvested for clinical diagnosis at the time of the routine follow-up, again in a blinded fashion (Graphical Abstract) (5, 18).

In total, for ACR grading agreement analysis, five distinctive datasets were used (Graphical Abstract; Supplementary Figure S2).

### Statistical Analysis

Baseline patient characteristics are expressed as means and standard deviations for normally distributed, and as median with interquartile range for non-normally distributed continuous variables. Categorical variables are expressed as counts and percentages.

Agreement between methods was assessed by weighted kappa, with weights being assigned to the grades of rejection according to how they would influence patient management (in terms of ACR: grades 2R and 3R carried increased magnitude of weight in the calculations) (19, 20). A *p*-value of < 0.05 was considered statistically significant. Statistical analysis was performed using STATA (Stata/IC 13.1 for Mac, Statacorp, Texas, United States).

## Results

### Patient Characteristics

The patient characteristics are shown in Supplementary Table S1. The majority were males, with mean age 54 ± 14 years, and median time from HTx of 24.6 months (IQR 4.9–35.6 months). Most of the patients were transplanted due to non-ischaemic dilated cardiomyopathy (52.2%), with arterial hypertension, hyperlipoproteinemia and diabetes mellitus as the most common comorbidities at the time of EMB harvesting (in 65.2%, 34.8% and 26.1% of the patients, respectively). Overall, the patients had normal left ventricular dimensions, preserved left-ventricular ejection fraction (LVEF) and did not have significant pulmonary hypertension. The most commonly used immunosuppressive regimen was the combination of mycophenolate with a calcineurin inhibitor. The clinical histopathological diagnoses are shown in Supplementary Table S2.

Detailed individual patient characteristics are shown in Supplementary Table S3.

### Agreement Between X-Ray Phase Contrast Imaging and Classical Histopathology

**FIGURE 1:**
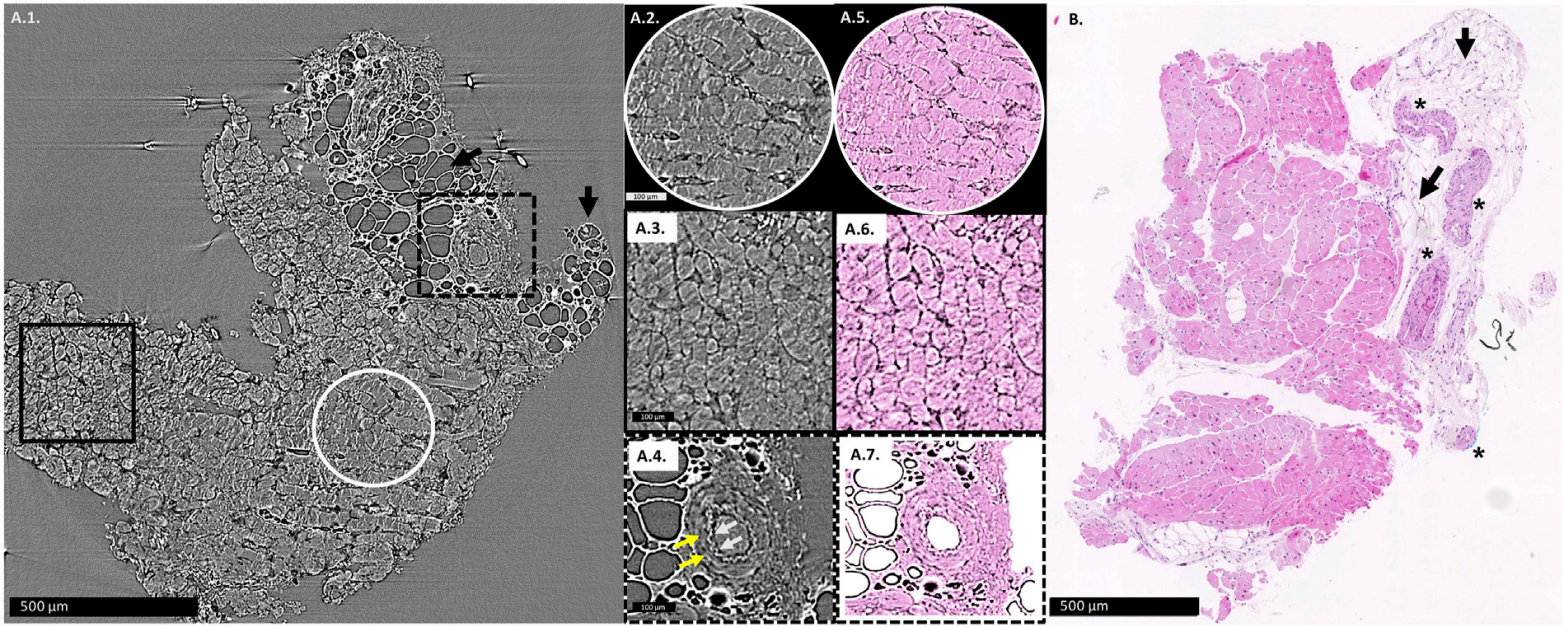
**(A1)**: X-PCI image of the EMB sample with no rejection (0R), where arteries, adipocytes (arrows) and cardiomyocytes can similarly be distinguished. Enlarged longitudinal cut of cardiomyocyte aggregates is shown in the white circle **(A2)**, while cardiomyocyte aggregates virtually transversely cut are shown in the black rectangle **(A3)**. **(A4)** Enlarged view of an artery, with white arrows showing the wavy contour of the tunica intima, while yellow arrows indicate the muscular tunica media. **(A5–A7)** the same X-PCI images shown in **(A2–A4)** digitally colorized to imitate H&E staining in classical histopathology. **(B)**: Histopathological slice of a similar area of the same sample, where arteries (*), adipocytes (arrows) and cardiomyocytes can be distinguished.

Figures 1A1–A4 show an example of an X-PCI image used in analysis, its digitally colored version (Figures 1A5–A7), and for comparison, the conventional histopathological image of the similar area of the same EMB sample is also shown (Figure 1B).

When using the clinical diagnosis made by classical histopathology as the reference method, X-PCI 2D and 3D virtual histopathology showed substantial agreement with the reference method [weighted kappa 0.80, (95% confidence interval: 0.43–1.16), and 0.73 (95% confidence interval: 0.35–1.12)] (Table 1). When using classical histopathology of the X-PCI imaged sample as the reference, a substantial agreement was achieved with both X-PCI 2D and 3D virtual histopathology as well [weighted kappa 0.80, (95% confidence interval: 0.42–1.17), and 0.73 (95% confidence interval: 0.34–1.11)]. The best agreement between the different methods was achieved in ruling out ACR that required treatment (Table 1).

**TABLE 1:** Agreement between different study datasets in the ACR grading according to the ISHLT 2004. Grading system. All the weighted kappa’s printed in bold have *p* < 0.01.

References vs. comparison method	Weighted kappa	95% CI
Clinical histopathological diagnosis vs.		
Study sample histopathology	**0.69**	0.27–1.10
X-PCI 2D virtual histopathology	**0.80**	0.43–1.16
X-PCI 3D virtual histopathology	**0.73**	0.35–1.12
Study sample histopathology vs.		
X-PCI 2D virtual histopathology	**0.80**	0.42–1.17
X-PCI 3D virtual histopathology	**0.73**	0.34–1.11
Clinical histopathological diagnosis by one observer vs.		
Clinical histopathological diagnosis	**0.93**	0.56–1.31
Study sample histopathology	**0.61**	0.26–0.97
X-PCI 2D virtual histopathology	**0.73**	0.38–1.08
X-PCI 3D virtual histopathology	**0.65**	0.30–1.0

When samples used for clinical diagnosis at the time of EMB were reassessed by the dedicated study pathologist, the agreement between the histopathological diagnosis made by this observer and the X-PCI 2D and 3D virtual histopathology remained substantial [weighted kappa 0.73, (95% confidence interval: 0.38–1.08), and 0.65 (95% confidence interval: 0.30–1.00)]. The agreement between the originally determined clinical diagnosis by classical histopathology (graded by different random pathologists) and the diagnoses reassessed by the study pathologist, i.e., inter-observer agreement was excellent [weighted kappa 0.93 (95% confidence interval: 0.56–1.31)].

**FIGURE 2:**
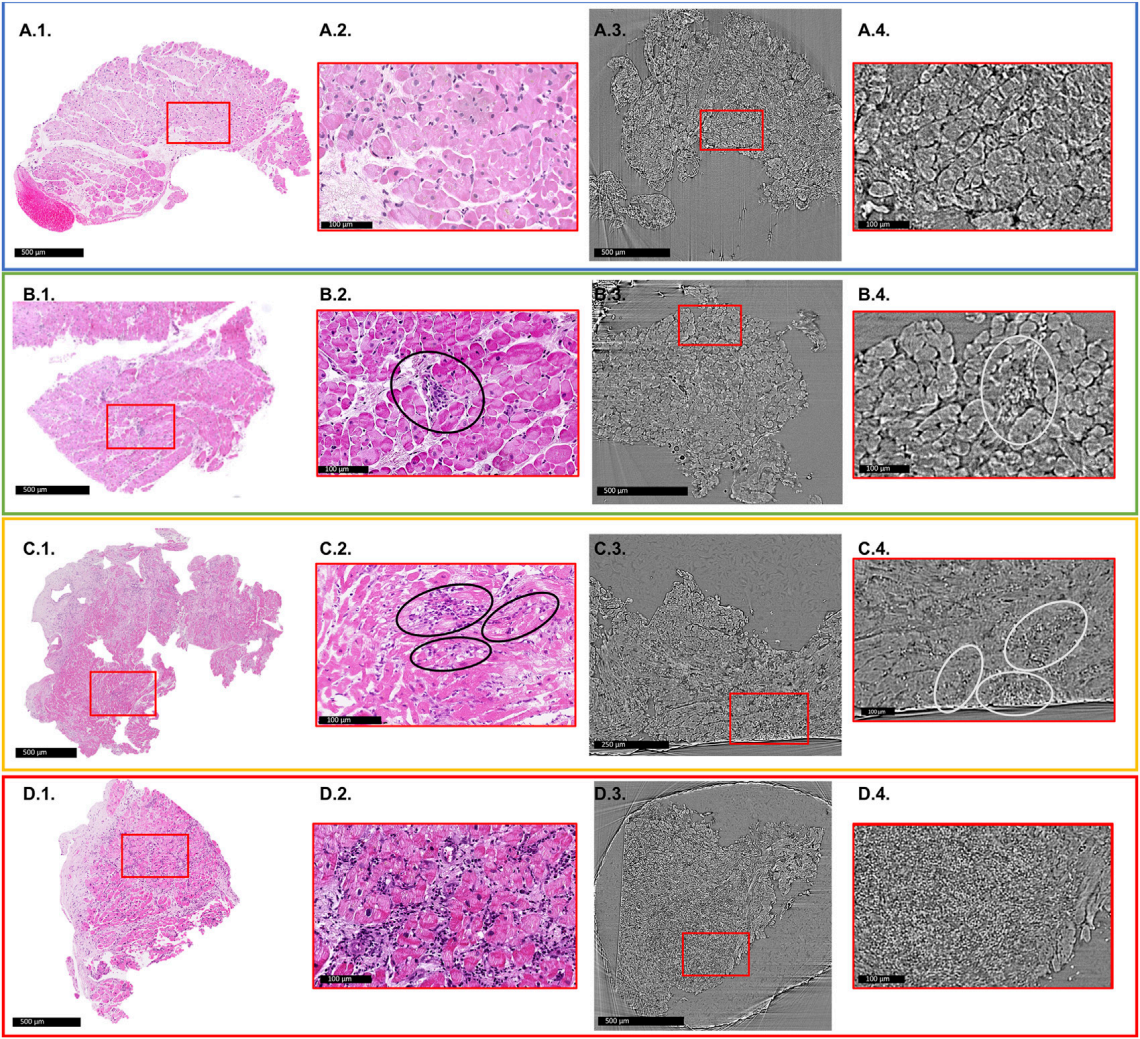
Comparison of histology slices with X-PCI images of a similar region of the same sample for each rejection grade. Red rectangles indicate areas of enlarged images shown in red boxes. **(A1–A4)** 0R grade. No cellular infiltrate present. **(A1,A2)** light microscopy; **(A3,A4)** XPCI. **(B1–B4)** 1R grade. Mild rejection (1R) is defined as cellular infiltrate with 1 area of cardiomyocyte injury. **(B2)** Black ellipse indicates area of perivascular cellular infiltrate, while in the **(B4)** area indicated with white ellipse shows interstitial infiltrate that replaces aggregates of cardiomyocytes indicating injury. This sample also shows signs of fibrosis most probably due to previous rejection episodes in this patient. **(C1–C4)** 2R grade. Moderate rejection is defined with two or more areas of cellular infiltrate with associated cardiomyocyte injury. **(C2)** Black ellipses show several interstitial infiltrates invading myocardium and replacing cardiomyocyte aggregates. The same is indicated in image **(C4)** with white ellipses. **(D1–D4)** 3R grade. Severe rejection is defined with diffuse cellular infiltration with diffuse damage to cardiomyocytes. **(D2)** shows extensive cellular infiltrates and diffuse disruption of tissue architecture. **(D4)** Cellular infiltrates completely replace the cardiomyocytes, and only a diffuse accumulation of cells is seen in this image.

Figure 2 shows an illustrative comparison between histopathology slices and X-PCI data of a similar area of the same sample for each of the different rejection grades.

## Discussion

In this study, we have used, for the first time, X-PCI to image human samples from EMBs. We have described the similarities between the features typical for normal myocardium and different stages of rejection, as well as inferences for its diagnostic use, as compared with routine assessment by clinical histopathology. We have shown that X-PCI allows for non-destructive visualization (effective isotropic pixel size of 0.65 µm) of the entire EMB sample in full-volume, allowing for the 3D dataset to be analyzed in any direction, without the use of staining agents. We have demonstrated that it is feasible for an experienced pathologist to successfully assess X-PCI images even for ACR grades, and with substantial agreement with classical histopathology.

Routine clinical follow-up protocols use EMB for the assessment of ACR by classical histology since no other method of structural tissue analysis has been proven as clinically relevant in the care of HTx recipients. Electron microscopy (EM) has been used for research purposes in patients with allograft rejection, and it did not add to the clinical decision process for these patients (21, 22). Conversely, EM is routinely used in the analysis of kidney biopsies, both for establishing the etiology of disease, as well as in surveillance of renal graft rejection (23). Although high-resolution and high-contrast optical confocal laser scanning microscopy (CM) provides substantial contrast, achieves axial resolution of around 800 nm, and allows for acquisition of 3D image datasets, it requires special fluorescence stains that may be unevenly distributed and hinder quantification attempts (24). A pilot-study by White et al. described and imaged features of ACR in human EMB samples of HTx recipients using optical CM, but did not compare ACR grading versus classical histopathology (25).

The latest studies performed with X-PCI show some clear advantages for tissue imaging: it is a non-destructive technique providing macro and/or micrometer scale details with high spatial resolution. Recently, possibilities of X-PCI for obtaining a comprehensive and 3D representation of a rat heart as a whole (organ level analysis at 5.8 um), but also at the cellular level were reported (cardiomyocyte analysis at 0.65 μm pixel size) (7, 8). Furthermore, X-PCI has successfully been utilized for the imaging of fetal human hearts in a similar fashion (9). It has thus been proven as a suitable technique to capture the morphology of the cardiac tissue, such as the organization of cardiomyocytes, vasculature, and collagen matrix.

Cardiomyocytes, interstitial spaces, cellular infiltrates, vascular structures, or adipocytes can easily be identified on X-PCI images (Figures 1, 2; Supplementary Figure S3). In addition, an experienced pathologist could discern the presence and extent of cellular infiltrate and its influence on tissue architecture, which is the basis of the ACR grading process (Figure 2). Propagation-based X-PCI images arranged according to recommendations for histopathological analysis of H&E sections (2D X-PCI virtual histology), yielded similar levels of agreement in ACR grading as was the case with 3D X-PCI virtual histology, in comparison to classical histopathology. Although we showed its potential in revealing features of ACR, it is important to understand that currently X-PCI cannot discern antibody mediated rejection (AMR), besides identification of cardiomyocyte destruction or architecture distortion.

Major benefit of 3D X-PCI virtual histology is its feature of providing detailed examination of the whole sample by producing dataset of several thousands of images.

In this study, the pathologist was provided with only one plane of the 3D structure to analyze the samples, but using simple open source imaging software such as Fiji (16, 17), one can virtually generate image slices in any desired direction, which could lead to an even better comprehension of the structural relations within the sample (Supplementary Figure S3; Supplementary Video S1).

The overall concordance between pathologists assessing ACR has been shown to be relatively low. One of the largest studies showed total agreement in ACR grading of only around 70%, and it was mainly based on concordance on samples with no signs of rejection (26). Indeed, the best agreement between methods in our study was achieved in ruling out ACR that required treatment. Moreover, a multicenter study on 827 EMBs showed the greatest variability in agreement between pathologists in grade 2 of the 1990 ISHLT grading system, having great importance since grade 2 and 3A (1R and 2R according to the 2004. ISHLT grading system) differ in treatment approach (26, 27).

Due to the obvious problems with the methodology, initiatives are under way to define ACR not solely on histopathology, but to combine it with clinical and laboratory parameters, or to completely change the paradigm by moving to the concept of “liquid biopsies” using the cell free DNA approach (4). However, classical histopathology remains the routine method worldwide. Besides integration of structural with clinical or laboratory data, one of the proposed directions to advance the area is switching to histopathology image digitalization, which is inherent to X-PCI.

## Limitations

At this point, X-PCI is a research tool confined to highly specialized synchrotron facilities, capable to image EMB samples at the required resolution and in a high throughput way. Nevertheless, major engineering advances are being undertaken to miniaturize the equipment in the translation of synchrotron techniques into laboratory-compatible setups, with the aim of their integration in the clinical setting (28–31). Therefore, parallel studies of the clinical feasibility and utility of this technique such as the one we present are needed in order to move forward this rapidly evolving field in an interdisciplinary manner.

The process of EMB grading with X-PCI at the synchrotron beam setup used in this study was dedicated to the identification of cellular infiltrates, currently not focusing on the identification of AMR, nor further analysis of cellular organelles such as nuclei. Furthermore, only one experienced pathologist was trained to grade the X-PCI samples.

This study has the limitation of its observational nature and small study sample, limiting its generalizibility, however still including the same ratio of high-grade to low-grade rejection samples as seen in everyday clinical practice. The study sample represents significantly more males, a trend that is also seen in the demographics of HTx recipients in large registries. However, based on these pivotal data, a full validation of the method additionally strengthened by a prospective design with larger study sample, and more observers, is foreseen in the future.

## Conclusion

We have demonstrated that X-ray based imaging, specifically exploiting the use of phase-contrast imaging, allows to evaluate ACR from EMBs. We compared X-PCI ACR grading with classical histopathology, showing a substantial agreement between the two methods. The main advantages of X-PCI include: 1) visualization of the whole EMB in a full-volume without need for slice selection, 2) a 3D dataset that can be analyzed in any direction, while 3) being a non-destructive method, not requiring any staining nor slicing (Table 2).

**TABLE 2:** Comparison of advantages and disadvantages of classical histopathology by light microscopy versus X-PCI virtual 2D/3D histopathology.

Classical histopathology by light microscopy	X-PCI virtual 2D/3D histopathology
Readily available in any pathology laboratory	Currently mainly limited to synchrotron facilities.
Destructive sample preparation	Sample remains intact.
Once already cut, the slice cannot be cut in different direction	No limitations in ways or number of times of reslicing.
Typically 10 or 20 slices	2,000+ slices (depending on the sample size).
Microtome slices of 3–4 um	Slice thickness in our setting 0.65 um.
Resolution limit around 0.2 um	Resolution limit ≈0.2 um. Pixel size was 0.65 um, but can be reduced (requires longer scan time).
Staining for basic structure differentiation	No contrast agents or stains used.
Cellular infiltrates may be characterized through specific staining	Cellular infiltrates identified, but can only be characterized from geometry or X-ray absorption coefficient resulting in grey level difference.
Vasculature assessment only in prepared slices	Vasculature assessment in the whole sample.

While the technique is currently mainly limited to synchrotrons, we do consider the study relevant for both a wider transplant and pathology community, since it introduces a non-destructive three-dimensional imaging method of tissue samples that could be utilized beyond heart biopsies. Ongoing developments should soon allow its transfer to hospital facilities, presenting X-PCI as a potential aid to routine clinical workflows.

We particularly highlight the potential of future translation of X-PCI from previous animal studies to a clinical application, thus setting the direction for future research to better understand the pathophysiological processes in the cardiac graft and its failure.

## Data Availability

The raw data supporting the conclusion of this article will be made available by the authors, without undue reservation.
