# Supplementary material for: A Novel Three-Dimensional Approach Towards Evaluating Endomyocardial Biopsies for Follow-Up After Heart Transplantation: X-Ray Phase Contrast Imaging and Its Agreement With Classical Histopathology
Source: Transpl Int. 2023 Jan 24;36:11046. doi: 10.3389/ti.2023.11046 (PMC9904361; doi:10.3389/ti.2023.11046)
Supplement: Supplementary file 1 [file DataSheet1.PDF]

Table of contents:

1. Supplementary Methods

2. Supplementary Results

3. References

4. Supplementary Figures

5. Supplementary Tables

6. Supplementary Video

1. Supplementary Methods

Acquired patient clinical data

Patient medical data were collected retrospectively from the electronic patient records, and included demographic data, relevant comorbidities, indication for HTx, specific post-transplant complications, laboratory parameters (estimated glomerular filtration rate (eGFR), high-sensitive troponin T (hsTnT), N-terminal pro-brain natriuretic peptide (NT-proBNP)), and immunosuppressive therapy at the time of the EMB (Table S1).

Endomyocardial biopsy

The endomyocardial biopsy was performed following a standardized clinical procedure in all the patients: a long vascular sheath was placed in the right or left femoral vein using the Seldinger technique, through which a myocardial bioptome (Cordis, Cardinal Health, Dublin, USA) was advanced under the fluoroscopic guidance into the right ventricle (RV). Endomyocardial biopsy samples were taken from the RV aspect of the interventricular septum. Myocardial sampling was performed according to the technical recommendations

proposed by the International Society for Heart and Lung Transplantation: at least 3-4 evaluable myocardial biopsy samples were obtained from different sites of the RV and the samples were further processed for histopathological diagnosis.(1) Besides the myocardial samples used for histopathological diagnosis in the clinical setting, an additional sample was taken for the purposes of this study. Classical histopathology was initially done on the 3-4 myocardial samples for routine clinical diagnosis of cellular cardiac allograft rejection grade, together with routine immunohistochemistry. In 20 patients, EMB samples were preserved only in a buffered formalin solution before analysis with X-PCI, while in 3 patients the samples were embedded in paraffin blocks prior to analysis. We have not seen any differences in quality of images or information gained when imaging formalin-fixed samples in comparison to samples embedded in paraffin blocks.

X-ray phase contrast imaging acquisition and visualization

This work has focused on the use of propagation-based X-PCI, which consists of a simple setup in which X-rays are left to propagate a certain distance between the sample and detector, so that interference patterns build up in tissue interfaces and contrast increases. Synchrotron radiation-based X-PCI acquisition was performed at the TOMCAT beamline (X02DA) of the Swiss Light Source (Paul Scherrer Institute, Switzerland). With no further tissue preparation, the samples were positioned in dedicated borosilicate glass tubes (2 mm diameter, 100 µm wall thickness) with degassed deionized water as medium in order to lower the chances of bubble and further artefacts formation. The tubes were then positioned on the sample stage for image acquisition with a X-PCI setup.(2) The samples were fully illuminated by a monochromatic X-ray beam with an energy of 20 keV. A propagation distance of 20 cm between sample and detector allowed for interference

patterns and edge-enhancement to form. At the detector, X-rays were converted to visible light with a LuAG:Ce 20 µm scintillator (Crytur, Czech Republic), amplified by a 10x objective, and recorded with an effective pixel size of 0.65 µm by a PCO.Edge 5.5 CMOS camera (PCO AG, Kelheim, Germany). Tomography was achieved by acquiring 2500 projections over 180°, which resulted in ~12 minutes acquisition time per volume. Reconstructed 3D datasets were obtained from projections using the Gridrec algorithm.(3) When samples were too large to fit in the field of view (1.66 x 1.66 x 1.4 mm^3), a maximum of 2 overlapping volumes were acquired and stitched using in-house developed Matlab scripts.

Images for Figure 1. were digitally colorized to mimic H&E staining images by creating and applying a linear MATLAB colormap from black to white, through the typical cell color in H&E. However, given the fact that cells and background have the same gray value, the background was manually colored white.

Histopathology

All the additional samples were embedded in paraffin prior to histopathological analysis. Paraffin blocks were sectioned with microtome into 4 µm thick slices which were stained by hematoxylin and eosin (H&E) and fixated on glass slides. At least 10 sections were analyzed by conventional light microscopy for histopathological diagnosis of CAR. (1)

3. Supplementary Results

Added value of X-PCI on the individual patient level - identification of Quilty lesions

A Quilty lesion is defined as nodular endocardial lymphocytic infiltrate, that sometimes may extend into the myocardium, potentially misleading the pathologist in recognizing it as a

cellular infiltrate damaging the myocardium. Accordingly, the EMB sample has to be cut in ample sections to try to assess the spatial relation of that infiltrate with the endocardium, which is seldom possible. (4,5)

Patient number 23 (Table S2, Table S3) was treated with intensive immunosuppressive therapy due to grade 2R rejection, as diagnosed by histopathology of the clinical EMB. At that time, ACR was only deemed present histopathologically; clinically, the patient had only moderately elevated NT-proBNP values, normal LVEF and mild pulmonary hypertension, all comparable to the previous clinical reports. Following that episode, the patient did not have repeated ACR episodes in the further course of treatment. When assessing the patient's virtual 2D and 3D histopathology for this study, it was only classified as 1R, similarly to the repeated histopathology done for this study (1R). The revision of all available images, triggered by the data obtained by 3D X-PCI, which showed the full extension of the infiltrates typical for Quilty lesions, concluded that the patient originally had only mild cellular rejection (Figure S1).

2. References

1. Stewart S, Winters GL, Fishbein MC et al. Revision of the 1990 Working Formulation for the Standardization of Nomenclature in the Diagnosis of Heart Rejection. J Heart Lung Transplant 2005;24:1710-20.
2. Dejea H, Garcia-Canadilla P, Cook AC, et al. Comprehensive analysis of animal models of cardiovascular disease using multiscale X-ray phase contrast tomography. Sci Rep 2019;9:6996.
3. Marone F, Stampanoni M. Regridding reconstruction algorithm for real-time tomographic imaging. J Synchrotron Radiat 2012;19:1029–37.
4. Marboe CC, Billingham M, Eisen H, Deng MC, Baron H, Mehra M, et al. Nodular endocardial infiltrates (Quilty lesions) cause significant variability in diagnosis of ISHLT grade 2 and 3A rejection in cardiac allograft recipients. *J Heart Lung Transplant*. 2005;24:S219-226.
5. Duong Van Huyen MF, Fedrigo M, Fishbein GA, Leone O, Neil D, Marboe C, et al. The XVth Banff Conference on Allograft Pathology the Banff Workshop Heart Report: Improving the diagnostic yield from endomyocardial biopsies and Quilty effect revisited. *Am J Transplant*. 2020;00:1–11.

3. Supplementary Figures.

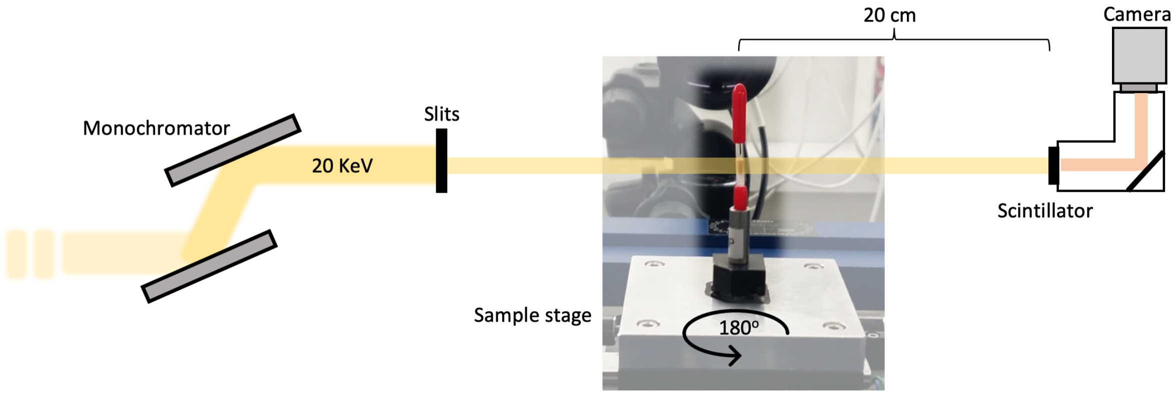

Figure S1. Scheme of the propagation-based X-PCI experimental setup for EMB samples at the TOMCAT beamline (Swiss Light Source). As shown in the picture, EMB samples are introduced in borosilicate glass tubes with degassed deionized water as medium. The yellow line represents the X-ray beam.

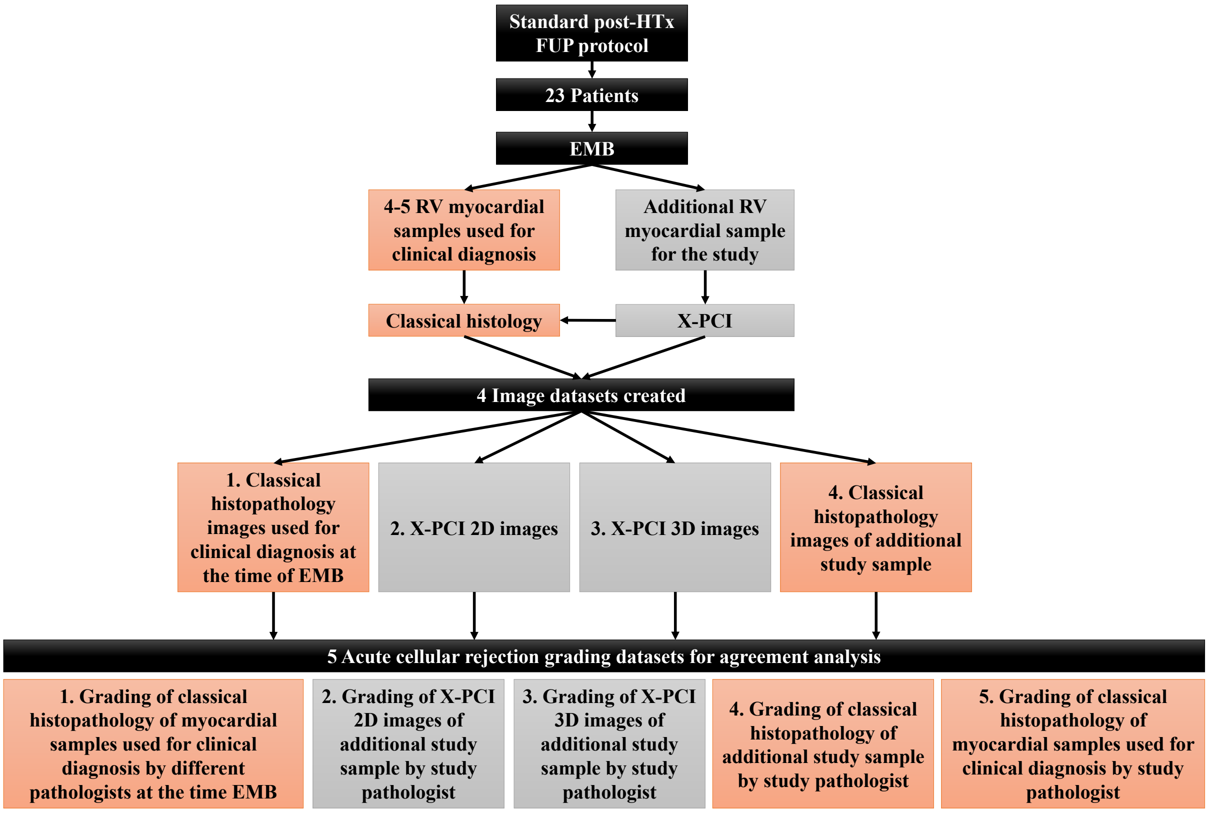

Figure S2. Research protocol. HTx- heart transplantation, EMB- endomyocardial biopsy, RV- right ventricle, X-PCI- X-ray phase contrast imaging, 2D- two-dimensional, 3D- three-dimensional, ACR- acute cellular rejection.

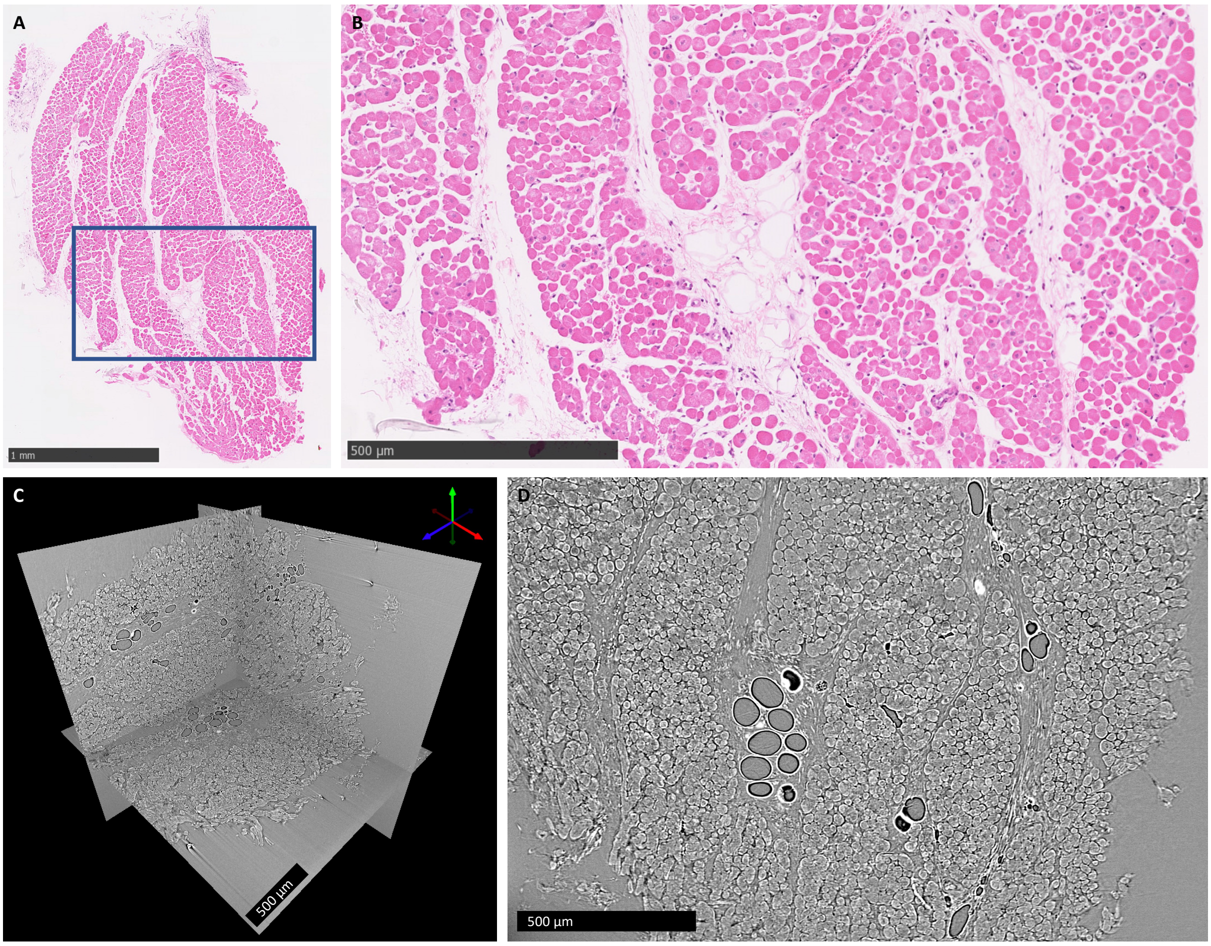

Figure S3. A: Classical histopathology slide of a 0R sample. The blue square marks the zoomed area in panel B. B: Zoomed in classical histopathology slide, where cardiomyocytes are clearly observed in cross-sectional direction. C: Three orthogonal slices representation of the X-PCI 3D dataset of the same, which shows that virtual navigation in any direction is possible. D: A specific reslice of the X-PCI 3D dataset that resembles the most the original histopathology image in B.

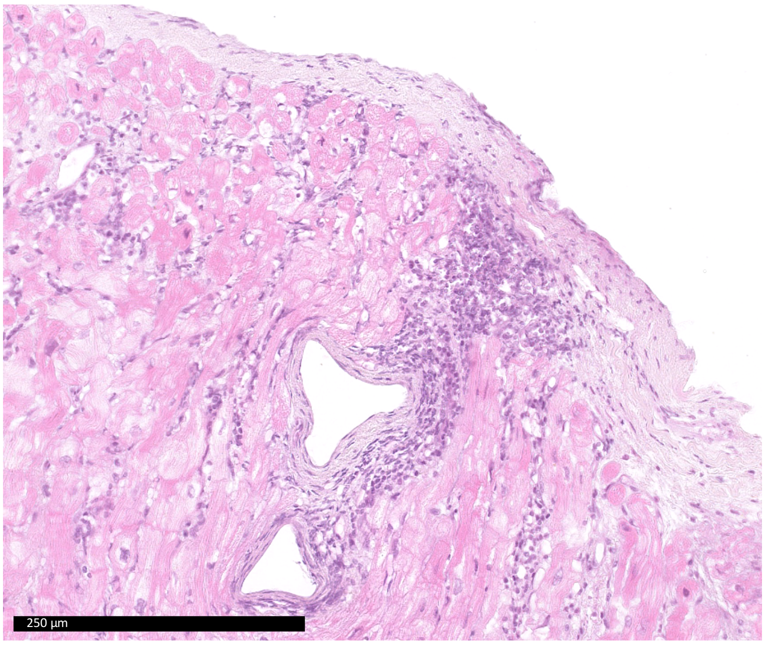

Figure S4. Histopathological H&E image of endomyocardial biopsy from the patient #23 indicating a Quilty lesion.

4. Supplementary Tables

Table S1. Characteristics of the patients at the time of EMB sampling. The values are represented as mean ± standard deviation or median (interquartile range).

**N**	**23**
**Males (n, %)**	**20 (86.9%)**
**Age (years)**	**54±14**
**Age at HTx (years)**	**52±14**
**Aetiology of heart failure (n, %)**	
- Dilated cardiomyopathy	12 (52.2%)
- Coronary artery disease	5 (21.8%)
- Arrhythmogenic cardiomyopathy	2 (8.7%)
- Restrictive cardiomyopathy	2 (8.7%)
- Congenital heart disease	1 (4.3%)
- Secondary cardiomyopathy	1 (4.3%)
**Time from HTx (months)**	**24.6 (4.9-35.6)**
**Previously treated rejection episodes (n, %)**	**3 (13%)**
**Vasculopathy (n, %)**	**2 (8.7%)**
**Comorbidities (n, %)**	
- Diabetes mellitus	6 (26.1%)
- Arterial hypertension	15 (65.2%)
- Hyperlipoproteinemia	8 (34.8%)
- Chronic kidney disease	5 (21.7%)
**eGFR (ml/min/1.73m^2)**	**66±21**
**hsTnT (ng/L)**	**21 (5-212)**
**NT-proBNP (pg/ml)**	**374 (123-10395)**
**Echocardiographic parameters**	
LVIDd (mm)	47.1±0.4
sPAP (mmHg)	30.5±9.9
LV EF (%)	62±8
**Immunosuppressive therapy**	
Steroid dose (mg)	10.5±8.8
Mycophenolate dose (g)	1.9±0.8
Tacrolimus (n, %)	11 (47.8%)
Cyclosporine (n, %)	12 (52.2%)
Everolimus (n, %)	1 (4.3%)

HTx - heart transplantation, eGFR - estimated Glomerular Filtration Rate, hsTnT - high sensitivity Troponin T, NT-proBNP - N-terminal pro-brain natriuretic peptide, LVIDd - Left ventricular internal diastolic diameter, sPAP - systolic pulmonary artery pressure, LV EF - left ventricular ejection fraction.

Table S2. Acute cellular rejection grades across different study datasets.

ID	Classical histopathology of myocardial samples used for clinical diagnosis by different pathologists at the time of EMB	X-PCI 2D virtual histopathology images of additional study sample by study pathologist	X-PCI 3D virtual histopathology of additional study sample by study pathologist	Classical histopathology of additional study sample by study pathologist	Classical histopathology of myocardial samples used for clinical diagnosis by study pathologist
**1**	0R	0R	1R	1R	0R
**2**	1R	1R	0R	1R	1R
**3**	0R	0R	0R	0R	0R
**4**	0R	0R	0R	1R	0R
**5**	1R	1R	1R	1R	1R
**6**	1R	0R	0R	0R	1R
**7**	0R	0R	0R	0R	0R
**8**	1R	0R	0R	*	1R
**9**	1R	0R	0R	1R	1R
**10**	1R	0R	0R	0R	1R
**11**	0R	0R	0R	0R	0R
**12**	0R	0R	0R	1R	0R
**13**	0R	0R	0R	0R	0R
**14**	0R	0R	0R	1R	0R
**15**	0R	0R	1R	0R	0R
**16**	0R	0R	0R	0R	0R
**17**	1R	0R	1R	0R	1R
**18**	0R	0R	0R	0R	0R
**19**	0R	0R	0R	1R	0R
**20**	1R	0R	0R	0R	1R
**21**	3R	3R	3R	3R	3R
**22**	2R	2R	2R	2R	1R
**23**	2R	1R	1R	1R	1R

*the sample was damaged during the transport and handling process following X-PCI imaging.

Pt.	Age at EMB (y)	Age at HTx (y)	Gender (M/F)	Time from HTx (m)	HTx indication	Previous Graft rejection episodes (0/1)	AMR/ DSA at EMB	CAV (0/1)	DM (0/1)	AH (0/1)	HLP (0/1)	CKD (0/1)	eGFR (ml/min /1.73m^2)	hsTnT (ng/L)	NTproBNP (pg/ml)	STR dose (g)	TAC (0/1)	CYS (0/1)	EVL (0/1)	MMF dose (g)	LVEF (%)	sPAP (mmHg)	LVIDd (cm)
**1**	54.6	53.9	M	9.0	NIDCM	0	0/0	0	0	1	1	0	94	12	123	12.5	0	1	0	2	65	40	4.6
**2**	50.5	50.3	M	3.4	ACM	0	0/0	0	0	0	1	0	71	36	660	15	1	0	0	2	65	20	4.8
**3**	59.9	59.7	M	1.9	RCM	0	0/-	0	1	1	1	0	60	94	977	25	0	1	0	3	65	34	5.6
**4**	64.4	56.1	M	100.2	NIDCM	1	0/0	0	1	1	0	1	56	6	243	5	1	0	0	2	65	20	4.7
**5**	59.9	58.9	M	12.2	NIDCM	0	0/0	0	0	1	0	0	60	10	365	10	0	1	0	2	65	30	5.5
**6**	62.3	62.3	M	0.8	CAD	0	0/0	0	0	0	0	0	60	138	2145	25	0	1	0	2	60	25	4.3
**7**	48.2	47.4	M	9.9	NIDCM	0	0/0	0	0	1	0	0	76	10	134	15	1	0	0	2	70	30	4.6
**8**	61.5	58.8	M	31.7	NIDCM	0	0/0	0	0	0	0	1	29	23	1868	2.5	1	0	1	0	60	20	5.2
**9**	61.9	59.6	M	27.6	NIDCM	0	0/1	0	1	1	0	0	60	15	2554	20	0	1	0	1.5	65	25	4.8
**10**	71.5	64.0	M	90.6	CAD	0	0/0	1	0	1	0	0	60	21	888	0	0	1	0	2.25	65	23	4.1
**11**	53.0	52.8	M	1.8	NIDCM	0	0/0	0	1	1	0	0	99	28	356	25	1	0	0	2	70	25	4.2
**12**	62.3	60.1	M	27.8	NIDCM	1	0/0	0	0	1	0	0	98	13	139	5	1	0	0	1	60	25	5.0
**13**	60.9	59.9	M	13.3	CAD	0	0/0	0	0	1	1	1	58	33	683	10	0	1	0	2	65	35	4.8
**14**	72.6	65.3	F	89.1	scCMP	0	0/-	0	0	1	1	0	74	5	374	0	0	1	0	0.75	-	-	-
**15**	46.2	45.9	M	4.4	ACM	0	0/0	0	0	0	0	0	71	27	327	15	1	0	0	3	60	25	5.2
**16**	63.9	61.9	M	24.6	NIDCM	0	0/0	0	0	1	0	0	60	28	462	5	0	1	0	1	65	20	4.5
**17**	46.9	44.8	M	25.5	NIDCM	0	0/0	0	0	0	0	1	28	58	195	5	1	0	0	1.5	65	40	4.3
**18**	33.3	30.8	M	31.3	RCM	0	0/1	0	0	0	0	0	60	12	158	5	1	0	0	2	65	30	4.5
**19**	64.6	64.2	M	4.1	CAD	0	0/0	0	0	1	1	0	60	14	236	17.5	0	1	0	1	60	30	4.5
**20**	64.4	60.1	M	52.3	CAD	0	0/0	0	1	1	1	0	60	13	147	0	0	1	0	3	60	25	5.0
**21**	18.3	15.1	F	39.5	NIDCM	0	1/1	0	0	0	0	1	45	212	10395	0	1	0	0	0.5	30	55	4.6
**22**	20.1	16.8	F	40.6	TOF-CHD	1	0/1	1	0	0	0	0	120	33	4593	2.5	1	0	0	1.5	50	50	4.1
**23**	47.2	46.8	M	5.4	NIDCM	0	0/0	0	1	1	1	0	60	17	708	15	0	1	0	3	70	45	4.7

Table S3. Additional demographic and clinical parameters of patients included in the study.
EMB: endomyocardial biopsy, HTx: heart transplantation, AMR- antibody-mediated rejection proven by endomyocardial biopsy, DSA- donor-specific antibodies (any mean fluorescence intensity), CAV: cardiac allograft vasculopathy, DM: diabetes melitus, AH: arterial hypertension, HLP: hyperlipoproteinemia, CKD: chronic kidney disease, eGFR: estimated glomerular filtration rate, hsTnT: high sensitive troponin T, NTproBNP: N-terminal pro brain natriuretic peptide, STR: steroid, TAC: tacrolimus, CYS: cyclosporine, EVL: everolimus, MMF: mycophenolate, NIDCM: non-ischaemic dilated cardiomyopathy, ACM: arrhythmogenic cardiomyopathy, RCM: restrictive cardiomyopathy, CAD: coronary artery disease, scDCM: secondary dilated cardiomyopathy, TOF-CHD: tetralogy of Fallot- congenital heart disease, LVEF: left ventricular ejection fraction, sPAP: systolic pulmonary arterial pressure, LVIDd: left ventricular internal dimension in diastole.

5. Supplementary Video

Video S1. Orthogonal slicing of an X-PCI 3D dataset of the endomyocardial biopsy. Virtual navigation through the sample is possible in any direction.
